# Multi-Modality Imaging in Vasculitis

**DOI:** 10.3390/diagnostics14080838

**Published:** 2024-04-18

**Authors:** Mohamed N. Allam, Nima Baba Ali, Ahmed K. Mahmoud, Isabel G. Scalia, Juan M. Farina, Mohammed Tiseer Abbas, Milagros Pereyra, Moaz A. Kamel, Kamal A. Awad, Yuxiang Wang, Timothy Barry, Steve S. Huang, Ba D. Nguyen, Ming Yang, Clinton E. Jokerst, Felipe Martinez, Chadi Ayoub, Reza Arsanjani

**Affiliations:** 1 Department of Cardiovascular Medicine, Mayo Clinic, Phoenix, AZ 85054, USA; allam.mohamed@mayo.edu (M.N.A.); abbas.mohammedtiseer@mayo.edu (M.T.A.);; 2 Department of Radiology, Mayo Clinic, Phoenix, AZ 85054, USAnguyen.ba@mayo.edu (B.D.N.);

**Keywords:** vasculitis, giant cell arteritis, Takayasu’s arteritis, polyarteritis nodosa, Kawasaki disease, granulomatosis with polyangiitis, eosinophilic granulomatosis with polyangiitis, Behçet’s disease, Cogan syndrome, immunoglobulin G—related disease, Doppler ultrasound, computed tomography, angiography, magnetic resonance imaging, positron emission tomography

## Abstract

Systemic vasculitides are a rare and complex group of diseases that can affect multiple organ systems. Clinically, presentation may be vague and non-specific and as such, diagnosis and subsequent management are challenging. These entities are typically classified by the size of vessel involved, including large-vessel vasculitis (giant cell arteritis, Takayasu’s arteritis, and clinically isolated aortitis), medium-vessel vasculitis (including polyarteritis nodosa and Kawasaki disease), and small-vessel vasculitis (granulomatosis with polyangiitis and eosinophilic granulomatosis with polyangiitis). There are also other systemic vasculitides that do not fit in to these categories, such as Behcet’s disease, Cogan syndrome, and IgG4-related disease. Advances in medical imaging modalities have revolutionized the approach to diagnosis of these diseases. Specifically, color Doppler ultrasound, computed tomography and angiography, magnetic resonance imaging, positron emission tomography, or invasive catheterization as indicated have become fundamental in the work up of any patient with suspected systemic or localized vasculitis. This review presents the key diagnostic imaging modalities and their clinical utility in the evaluation of systemic vasculitis.

## 1. Introduction

**Figure 1:**
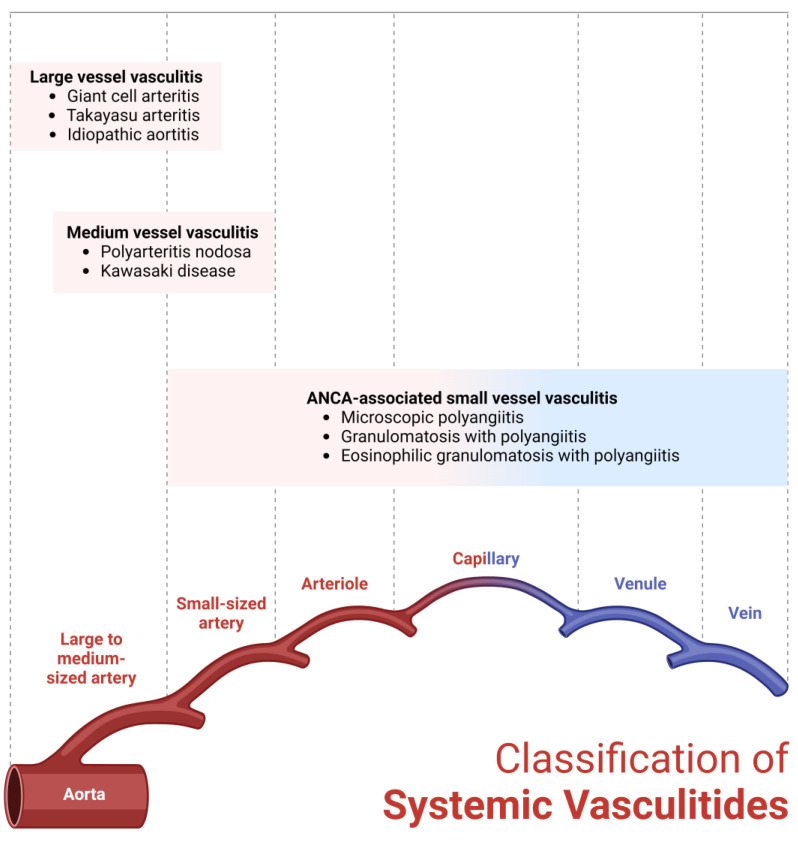
Classification of systemic vasculitides. Created with Biorender.com.

Systemic vasculitides represent a group of rare inflammatory conditions characterized by leukocyte infiltration in the blood vessel wall. They are classified into large-, medium-, and small-vessel vasculitis, based on the predominant size of the vessels involved, although there can be overlap between the different categories. The acute phase is usually associated with an extravascular systemic inflammatory reaction responsible for the systemic symptoms of the disease and marked by elevation of acute inflammatory markers including erythrocyte sedimentation rate (ESR) and C-reactive protein (CRP) [1]. Systemic vasculitides can be associated with serious adverse outcomes without prompt diagnosis and early use of immunosuppressive agents. Recent advances in the field of vascular imaging have revolutionized the diagnosis of the different vasculitic disorders as well as detecting the extent of the disease, complications, and monitoring therapy, especially when biopsy is not feasible. In this review, we will discuss the use of multi-modality imaging in the diagnosis and management of vasculitis and evaluate their characteristic radiological findings (Figure 1).

## 2. Classification of Vasculitides

### 2.1. Large-Vesel Vasculitis

#### 2.1.1. Giant Cell Arteritis

Giant cell arteritis (GCA) is the most common systemic vasculitis with a higher incidence among older individuals of North European descent [2]. Infiltration of the large and/or medium-vessel wall by macrophages and subsequent formation of giant cells and granulomatous inflammation is the hallmark of the disease, hence the name “GCA” [3]. Persistent inflammation eventually leads to progressive intimal thickening and transmural damage, resulting in ischemia and aneurysm formation.

GCA tends to involve the cranial branches of the carotid arteries (cranial GCA), causing cranial manifestations such as headache, visual disturbances, scalp tenderness, and jaw claudication. Ophthalmic involvement is considered a medical emergency and high-dose intravenous pulse steroids should be initiated immediately [4]. Extra-cranial large-vessel disease, including the aorta and its main branches, occurs in almost 80% of the patients and has a clinical impact on disease prognosis and rate of relapse [5,6,7]. Patients with large vessel GCA (LV-GCA) are typically younger (66 years compared to 72 years in those with cranial GCA) and mostly women (83%) [8]. LV-GCA mainly involves the subclavian arteries (75%), the aorta (50%), and the femoral arteries (30–40%) [8]. Polymyalgia rheumatica (PMR) is observed in 40–60% of patients with GCA and manifests as shoulder and hip joint pain [9].

Temporal artery biopsy has been the gold standard diagnostic test for disease confirmation. However, patients with predominantly large-vessel involvement will typically have a negative temporal artery biopsy. Moreover, the patchy pattern of the disease, early glucocorticoid use, and the bilaterality and length of the sample can all affect the diagnostic sensitivity of temporal artery biopsy [10]. Hence, clinical imaging plays an important role in the diagnosis of GCA, especially in detecting large-vessel disease. In fact, imaging of the aorta and its major branches is recommended in all patients with GCA (Table 1) [11].

Ultrasound imaging has been widely used in the diagnosis of large vessel vasculitis (LVV) including GCA. Due to the availability of high-frequency transducers (>15 MHz), color Doppler ultrasound (CDUS) can image the temporal arteries with an excellent resolution. In the 2022 American College of Rheumatology (ACR)/European Alliance of Associations for Rheumatology (EULAR) GCA Classification Criteria, demonstrating a non-compressible homogenous hypoechoic wall thickening on ultrasound as a sign for mural edema “the halo sign”, has dominated a proposed model for GCA diagnosis as the strongest predictor for GCA, besides temporal artery biopsy [12]. A meta-analysis which included eight studies comparing ultrasound to the clinical diagnosis of cranial GCA as a reference, has yielded a pooled sensitivity of 77% and a specificity of 96% [7,13,14,15,16,17,18,19,20].

Ultrasound may also demonstrate halos in the axillary, subclavian, vertebral, and carotid arteries. The addition of bilateral axillary arteries to temporal artery ultrasound may improve the overall accuracy of the test. This could be related to the longer time for resolution of inflammation-related changes in these larger vessels, compared to temporal arteries, especially in cases when glucocorticoids have already been initiated [21]. CDUS may also show arterial stenosis as a complication from GCA by detecting changes in the flow velocities profile of the vessel [22].

Ultrasound has several advantages, including but not limited to its wide availability, low cost and absence of radiation. Due to comparable sensitivity and specificity, CDUS has already surpassed temporal artery biopsy as the diagnostic test of choice in some centers [20,23]. Currently, the 2018 EULAR guidelines recommend temporal artery ultrasound as the first diagnostic modality in patients with suspected cranial GCA [11]. However, the 2021 ACR/Vasculitis Foundation (VF) guidelines still recommend unilateral long segment (>1 cm) temporal artery biopsy over ultrasound due to United States radiologists having less experience with ultrasound compared to their colleagues in Europe [4].

**Figure 2:**
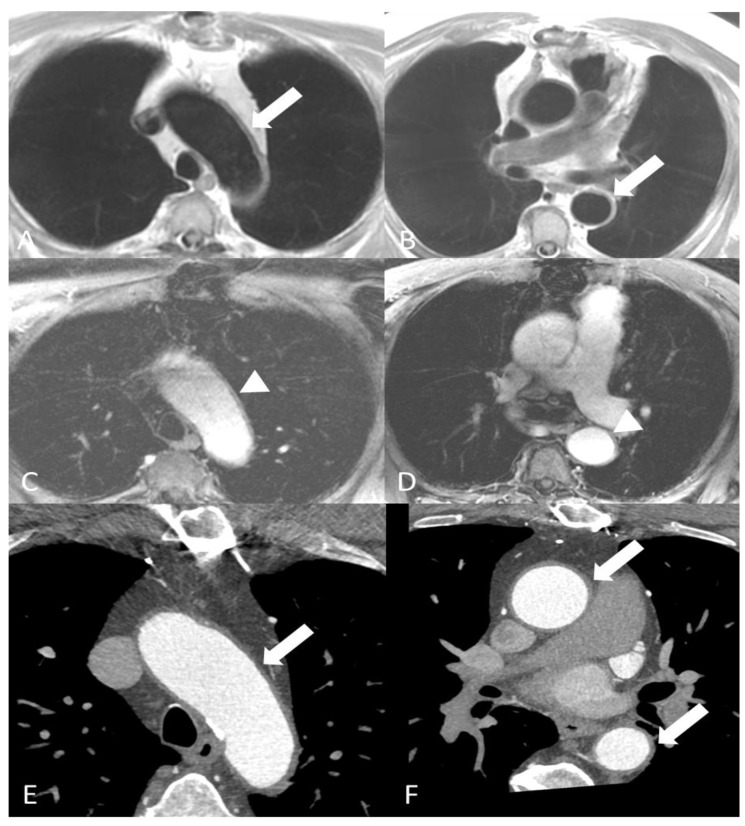
A 75-year-old man with known giant cell arteritis. Axial double inversion recovery MR images (**A**,**B**) demonstrating concentric mural thickening of the thoracic aorta (arrows). Axial T1-weighted images after intravenous contrast administration (**C**,**D**) demonstrating mild enhancement to the aortic wall more pronounced in the arch and descending aorta (arrowheads), consistent with active vasculitis. Images of chest CTA (**E**,**F**) showing concentric mural thickening of the ascending aorta, aortic arch and descending aorta (arrows).

Magnetic resonance imaging (MRI)/magnetic resonance angiography (MRA) can be used to identify GCA in both cranial arteries and large vessels. It can demonstrate mural thickening, and the presence of gadolinium-enhancement of the affected arterial walls on ECG-gated high-resolution T1 weighted phase with fat suppression can suggest active inflammation [24] (Figure 2A–D).

In a large systematic review, MRA had a pooled sensitivity of 73% and specificity of 88% when compared to clinical diagnosis of cranial GCA [7]. The same study indirectly showed MRA to have a higher sensitivity than ultrasound (93% vs. 70%) when both were compared to temporal artery biopsy. However, a retrospective study directly comparing both of them has shown similar sensitivity and specificity (67% and 91%, respectively) [25]. A prospective study comparing both techniques is still not available. The 2018 EULAR guidelines recommend MRI of the cranial vessels to be used as an alternative imaging modality for GCA diagnosis if ultrasound is inconclusive or not available [11].

The advantages of MRI/MRA are the lack of radiation and the ability to depict wall inflammation and disease complications with a high resolution. However, availability, cost, claustrophobia, and the presence of certain hardware in the body can limit the use of this modality. Gadolinium may cause nephrogenic systemic fibrosis (NSF) in patients with advanced renal insufficiency, although the associated risk with newer gadolinium-based agents is uncertain and may be lower [26].

Computed tomography angiography (CTA) is widely used in large vessel vasculitis due to its high availability and excellent spatial and temporal resolution. A biphasic imaging acquisition, including non-contrast and EKG-gated arterial phases, is commonly used with the ability to reconstruct three-dimensional images of the aorta and its main branches [27]. CTA may show the early changes that occur in vasculitis such as concentric wall thickening, wall enhancement, and perivascular fat stranding (Figure 2E,F). Wall thickening can be differentiated from mural hematoma in the unenhanced phase [28]. If a delayed phase is acquired, enhancement of the adventitia can be accentuated in contrast to the edematous intima causing the “double ring sign”.

CTA can also demonstrate late complications including stenosis, dissection, or aneurysms. In a prospective study, the sensitivity and specificity of CTA in the diagnosis of GCA were 73% and 78%, respectively, compared to clinical diagnosis as a reference. However, the accuracy of the test may have been underestimated due to the small number of patients enrolled in the study and lack of power [29]. ACR/VF guidelines conditionally recommend CTA or MRI/MRA in all patients newly diagnosed with GCA to assess for large vessel involvement [4].

The advantages of CTA include the wide accessibility and the ability to assess the aorta and its main branches in a single acquisition with high resolution and short scan time. Additionally, a coronary CTA can be obtained in the same scan if there is a suspicion of coronary artery disease. CTA is also able to differentiate between vasculitis and atherosclerosis, which is common in elderly patients with GCA. CTA is not recommended for the assessment of small vessels (less than 0.2 mm), including cranial arteries [11]. The differentiation of active wall inflammation from fibrosis or remodeling can be challenging with CTA, as well as MRA. Additionally, radiation exposure and the risk of kidney injury and allergic reactions associated with iodine-based contrast can be limiting for some.

**Figure 3:**
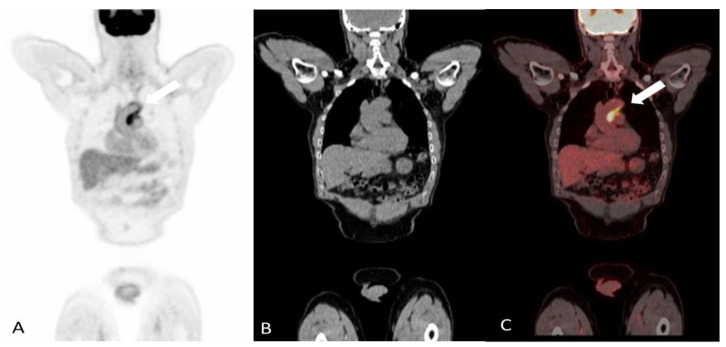
A 60-year-old woman with giant cell arteritis. Coronal images of attenuation corrected F-18 FDG PET (**A**), CT without contrast (**B**) and fused PET/CT (**C**) demonstrating moderate intensity linear increased tracer activity in the wall of the ascending aorta and proximal arch (arrows), most consistent with active vasculitis.

**Figure 4:**
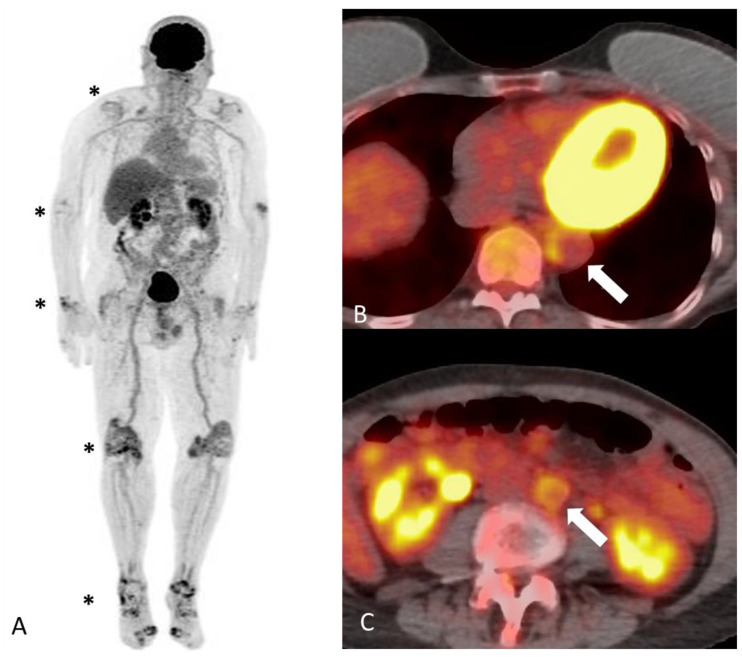
A 78-year-old man with polymyalgia rheumatica and active vasculitis. Maximum intensity projection images of FDG PET (**A**) demonstrating abnormal radiotracer accumulation in multiple joints (shoulders, elbows, hands, hips, knees and feet) in keeping with polyarticular inflammatory arthropathy (asterisks). Axial images of fused PET CT at the level of the descending aorta (**B**) and abdominal aorta (**C**) showing near concentric tracer avid mural thickening to the aorta (arrows), most consistent with active vasculitis.

^18^Ffluorodeoxyglucose positron emission tomography/computed tomography (FDG-PET/CT) is a key whole-body imaging modality in the field of cancer and infectious diseases, and now has gained increasing utilization in the diagnosis of various inflammatory conditions including large-vessel vasculitis. The FDG radiotracer accumulates in the high metabolizing inflammatory cells in areas of active inflammation and is subsequently phosphorylated to FDG-6-phosphate. Cells are unable to further metabolize FDG-6-phosphate preventing it from leaving the cells. The radioactive fluorine in the FDG eventually decays via beta decay, and the emitted positron is detected by the PET scanner. Concurrent low-dose CT provides attenuation correction and facilitates anatomic localization [30]. PET/CT has an advantage over other imaging modalities in visualizing active inflammation in otherwise structurally normal vessels and helps characterize disease extent in GCA and coexisting PMR [31] (Figure 3 and Figure 4). Large vessel FDG uptake equal to or higher than liver uptake (as a reference) is considered a strong criterion for active inflammation; PETVAS (PET Vascular Activity Score) is a composite score developed to allow for quantitative grading of disease burden with minimal interobserver variability. However, PET interpretation remains largely qualitative in many centers [32].

In a meta-analysis that included four studies, PET/CT had a pooled sensitivity of 90% and specificity of 98% to detect large-vessel inflammation in LV-GCA when compared to controls [33]. PET/CT may also be used to monitor treatment [34]. A recent study showed that normalization of FDG uptake during treatment correlates well with clinical improvement and reduction in inflammatory markers, compared to MRI [35].

Cost and radiation exposure may be a disadvantage for PET/CT. However, radiation exposure is much lower with newer generation scanners and comparable to CT examination with contrast, and a whole body FDG-PET/CT may have lower radiation burden. PET is not widely recommended for the assessment of cranial vessels due the lack of evidence and proximity to the brain, making it a challenge to distinguish vessels from brain uptake [11]. Additionally, glucocorticoid treatment attenuates FDG vascular uptake in LV-GCA, particularly after 10 days of treatment, and may lower the sensitivity of the test [36]. On the other hand, atherosclerosis may overestimate the degree of inflammation in elderly patients, mainly at the level of abdominal aorta and iliac arteries [37].

Combined FDG-PET/MRI is an evolving hybrid technique with a lower radiation exposure compared to PET/CT and may be an attractive alternative when frequent follow-ups are needed [38].

Heart involvement including coronary arteritis, aortic insufficiency, exudative pericarditis, or myocarditis is rare in GCA [39]. Echocardiography and cardiac MRI may be used to evaluate for myocardial or pericardial complications if clinically warranted.

#### 2.1.2. Takayasu’s Arteritis

Takayasu’s arteritis (TAK) is a rare large-vessel vasculitis that primarily affects the aorta and its main branches, and the pulmonary arteries. TAK mainly affects women younger than 40 years. It is more prevalent in individuals of Asian ancestry [2]. In the USA, the prevalence of TAK was reported as 8.4 cases per million people in a population-based study [40].

T-lymphocyte-mediated inflammation in the vessel wall is thought to be responsible for the vascular injury in TAK, causing inflammatory infiltration of the adventitia and media layers and hyperplasia of the intima [41]. A genetic component may also play a role in the pathogenesis of TAK, although it is not fully elucidated [42].

Patients may report nonspecific constitutional symptoms in the early phase of the disease, including fatigue and weight loss. Not uncommonly, patients may initially present with symptoms related to late complications including limb claudication, stroke, weak peripheral pulses, and blood pressure differences between extremities. Secondary hypertension occurs in more than 50% of the patients due to the involvement of one of the renal arteries or both. In these cases, blood pressure measurement can be misleading if there is disease in the arteries of the arms, and blood pressure from an unaffected thigh should be obtained [43]. Coronary arteries can be involved in about one-third of TAK patients with a risk for acute coronary syndrome (ACS) [44,45]. The demographic and clinical context coupled with elevated inflammatory markers should raise suspicion for TAK. However, imaging is mandatory to establish a diagnosis and determine the extent of the disease.

CDUS can be used to evaluate the subclavian and carotid arteries and will show findings similar to GCA, including halos in the arterial walls and changes in blood Doppler velocities. Concentric, moderately echogenic homogenous thickening of the wall of the common carotid artery is termed the “macaroni sign” and is highly specific for TAK [46,47]. In a systematic review and meta-analysis, the pooled sensitivity and specificity of ultrasound in TAK are 81% and almost 100%, respectively [48]. Contrast-enhanced ultrasound (CEUS) using intravenous injection of microbubbles has been suggested to improve imaging quality. Contrast enhancement within the arterial wall may indicate increased microvasculature due to active inflammation [49]. Recently, ultrasound localization microscopy (ULM)—using ultrafast ultrasound—has been used to quantify microbubbles circulating in the proliferating vasa vasorum in the inflamed walls to help determine disease activity more objectively [50]. It is important to note that ultrasound is of limited value for imaging of deeper vessels such as the thoracic aorta.

MRI/MRA is the imaging modality of choice in TAK. It can provide a full arterial survey of the body without radiation exposure; this is of particular importance when regular imaging surveillance is needed in young women after treatment. MRI/MRA can demonstrate mural thickening similar to LV-GCA, and gadolinium enhancement in the arterial wall is suggestive of acute inflammation. MRI has a sensitivity and specificity of 98% and 100%, respectively, when referenced to invasive angiography [51].

**Figure 5:**
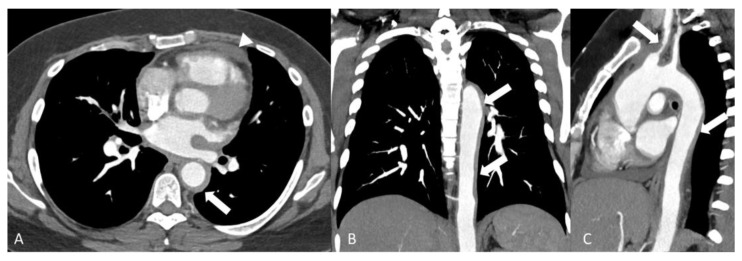
A 28-year-old man with active Takayasu’s disease. Axial (**A**), coronal (**B**) and sagittal (**C**) images of chest CTA demonstrating concentric mural thickening involving the ascending aorta, arch, descending aorta and proximal right brachiocephalic artery (arrows) and small reactive pericardial effusion (arrowhead).

**Figure 6:**
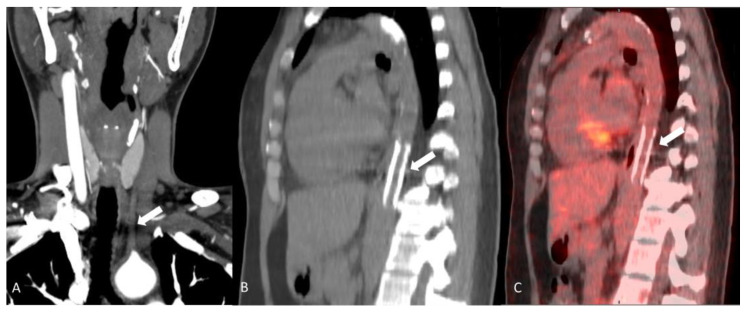
A 32-year-old woman with history of Takayasu’s disease. Coronal maximum intensity projection image of neck CTA (**A**) showing complete occlusion to the left common carotid artery (arrow). Sagittal images of CT without contrast (**B**) and F-18 FDG PET/CT (**C**) demonstrating distal descending aorta stent (arrows) with no pathological abdominal wall radiotracer accumulation consistent with sequela of chronic arteritis with no evidence of acute inflammation.

CT angiography can show similar findings to MRI including thickening and smoothly tapered narrowing of the vessel lumen with excellent sensitivity and specificity (>90% for both) [48] (Figure 5). It is also able to identify late complications including artery stenosis and aneurysmal dilation (Figure 6). Head CT (HCT) and CTA of the head and neck are essential for the evaluation of TAK patients presenting with acute stroke-like symptoms.

FDG-PET/CT is widely utilized in TAK and shows findings similar to LV-GCA with comparable accuracy (Figure 6). It was also shown to have a pooled sensitivity of 81% and specificity of 74% for disease activity, compared to clinical assessment. However, the heterogeneity between the included studies was significant, mainly due to the lack of consensus on how to quantify disease activity [48]. Therefore, the clinical utility of PET in measuring disease activity is still under investigation.

**Figure 7:**
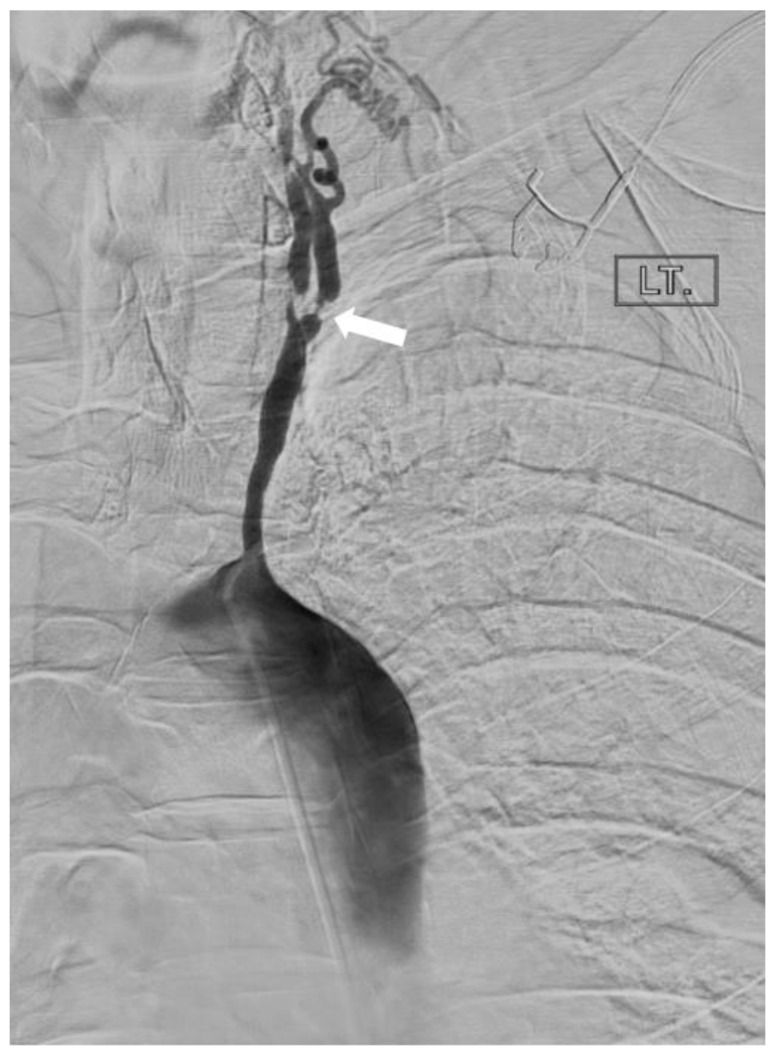
A 54-year-old woman with history of Takayasu’s disease. Invasive catheterization with injection of contrast in the aorta at the origin of the left (bovine) common carotid artery; stenoses at the origin of the left internal and external carotid arteries are demonstrated (arrow), and the collateral vessels that they give rise to. The right common carotid and right subclavian arteries are completely occluded. The left vertebral and left subclavian arteries are occluded.

Overall, the 2021 ACR/VF guidelines recommend CTA, MRA, or FDG-PET/CT in all patients with suspected TAK [4]. The 2018 EULAR guidelines recommend MRI/MRA as the first line imaging technique in the diagnosis of TAK, with PET, CTA, and/or ultrasound to be used as alternatives [11]. In both guidelines, invasive angiography is no longer required for diagnosis of TAK unless surgical planning or central blood pressure measurement is needed (Figure 7).

#### 2.1.3. Clinically Isolated Aortitis (Idiopathic Aortitis)

Clinically isolated aortitis (CIA) has been described as a single-organ vasculitis of the ascending aorta, in the absence of any demographic or clinical characteristics suggestive of a systemic inflammatory disease or an infectious process. The diagnosis is usually made as an incidental imaging finding, or on tissue examination of a surgically resected aneurysm. The histopathology of CIA is similar to GCA with granulomatous inflammation in the vessel wall, although a lymphoplasmacytic pattern may also be seen [52].

In a nationwide Danish population-based study, 6.1% of resected thoracic aortic aneurysms had aortitis, and three-quarters of them were CIA [53]. In North America, the annual incidence of CIA is reported as 8.9 per million individuals over the age of 50 years [54]. Age and a history of connective tissue disorder have been identified as risk factors [55].

MRA and CTA may show adventitial and/or periaortic thickening due to inflammation of the periaortic fat and the surrounding soft tissue (periaortitis) [56]. PET-CT may play a role in determining disease activity [35]. MRA, CTA, or PET-CT can all be used to image other vascular territories and rule out any other vascular involvement suggestive of an undiagnosed systemic disease. Patients with CIA are at risk of subsequent dissection or aneurysm formation, and following these patients with surveillance imaging is recommended [57]. Currently, there are no specific diagnostic criteria for CIA, and more research is warranted to better understand its clinical outcomes [1,54].

### 2.2. Medium-Vessel Vasculitis

#### 2.2.1. Polyarteritis Nodosa

Polyarteritis nodosa (PAN) is an antineutrophil cytoplasmic antibodies (ANCA) negative medium-vessel vasculitis with additional involvement of small vessels. It can affect any organ of the body except the lungs [58]. The incidence rate of PAN is 4.4–9.7 per million in Europe [59]. Men are more affected than women at a 1.5:1 ratio, typically at age of 40–60 years [2]. Most cases of PAN are idiopathic; however, PAN may occur secondary to hepatitis B virus, hepatitis C virus, and certain types of leukemia [58,60]. In fact, the prevalence of PAN has significantly decreased due to the wide availability of the Hepatitis B vaccine [61].

The necrotizing arteritis in PAN initially causes arterial lumen narrowing, and eventually will lead to ischemia and formation of aneurysms [62]. Patients present with constitutional symptoms in 90% of cases, in addition to organ-specific manifestations. Involvement of the skin and peripheral nervous system is common and includes ulcers, purpura, livedo reticularis, and mononeuritis multiplex. The kidneys are the most commonly involved internal organ. PAN can cause silent renal infarcts or manifest as renal insufficiency and hypertension [63]. Also, rupture of a renal microaneurysm can cause perirenal hematomas. Abdominal angina can occur due to mesenteric ischemia [64]. Involvement of the coronary arteries has been also described. However, overt myocardial infarction is uncommon [39].

**Figure 8:**
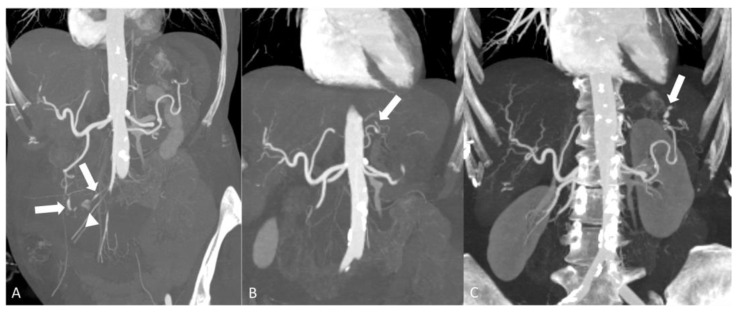
An 80-year-old woman with polyarteritis nodosa presenting with gastrointestinal bleeding. Coronal maximum intensity projection images of abdomen CTA at three different levels demonstrating pseudoaneurysms of the celiac and superior mesenteric artery branches secondary to arteritis. (**A**) Pseudoaneurysm of the gastroduodenal artery and jejunal branch of the superior mesenteric artery (arrow) and contrast extravasation into the proximal small bowel loop consistent with active GI bleed (arrowhead). (**B**) Pseudoaneurysm of the left gastric artery (arrow). (**C**) Pseudoaneurysm of the distal aspect of the splenic artery (arrow).

Invasive angiography is the gold standard test to diagnose visceral PAN by showing the characteristic microaneurysms of the disease. Biopsy should be obtained in cases with cutaneous and muscle involvement, as these tissues are more easily accessible [65]. Modern advances in noninvasive imaging permit CTA and MRA to screen for visceral PAN, owing to enhanced image resolution. Additionally, noninvasive imaging has the advantage of assessing the visceral parenchyma. CTA is able to image the more distal branches of the mesenteric arteries better than MRA, and may show microaneurysms in the visceral arteries as well as extravasation due to rupture of a microaneurysm [66] (Figure 8). CTA can also be used to evaluate for coronary involvement. PET/CT may show an increased uptake in the involved organs especially in the legs [67]. The 2021 ACR/VF guidelines conditionally recommend the initial use of abdominal vascular imaging with CTA or MRA, and escalating to conventional angiography only if imaging results are negative and there is high suspicion for abdominal involvement [68].

#### 2.2.2. Kawasaki Disease

Kawasaki disease (KD) is a self-limiting vasculitic disease that mainly affects the medium-sized arteries, especially the coronary arteries [1]. It is the second most common vasculitis in children, after immunoglobulin A vasculitis. The disease is more common in children of Asian ancestry, and boys are more affected than girls [69]. In the United States, the hospitalization rate of Kawasaki disease is 19 per 10,000 children younger than 5 years [70]. KD may rarely affect adults, especially in HIV-infected patients [71,72].

In KD, a neutrophilic infiltration of the arterial wall causes destruction of the medial smooth muscles and the elastic lamina with subsequent formation of aneurysms. The etiology of KD remains unknown although genetic factors appear to have a predisposing role in the pathogenesis [73,74]. Children initially present with fever and signs of mucocutaneous inflammation, including conjunctivitis, mucositis, rash, lymphadenopathy, and edema of the dorsum of their hands and feet [75].

KD affects the coronary arteries in up to 25% of cases in the form of ectatic, saccular or fusiform aneurysms, and children with KD are at risk for serious cardiovascular complications [76]. Risk factors associated with coronary artery aneurysms include male gender, age less than one year, late diagnosis, and delayed treatment with intravenous immunoglobulin [77]. The size of the coronary artery aneurysms (CAA) correlates with future complications, including rupture, thrombosis, and stenosis with subsequent myocardial infarction and death [75,78]. On the other hand, peripheral aneurysms in KD tend to regress over time [79].

**Figure 9:**
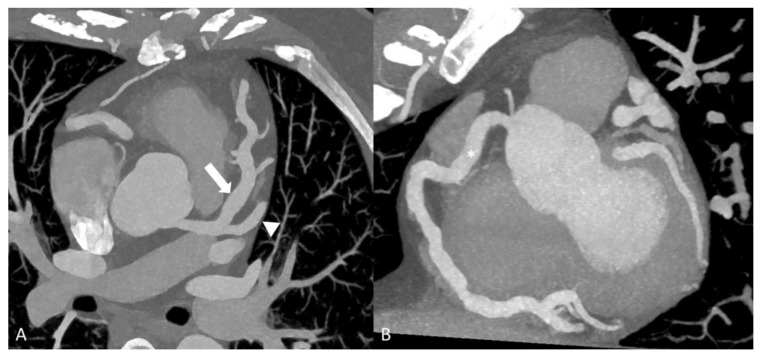
A 35-year-old woman with Kawasaki’s disease. Double oblique MPR images of coronary CTA (**A**,**B**) showing diffuse aneurysmal dilation to the LAD (arrow), large caliber first diagonal branch (arrowhead) and RCA (asterisk).

A transthoracic echocardiogram (TTE) should be performed early in the disease to obtain a baseline status of left ventricular systolic function and assess the proximal coronary arteries in young children for future monitoring. However, the sensitivity of TTE in visualizing the coronary arteries in older children and adults is very limited [75]. In this setting, coronary CTA (C-CTA) or MRA can be used to assess for coronary artery abnormalities through to the very distal branches with high resolution (Figure 9) [80,81]. Cardiac MRI may show late gadolinium enhancement in areas affected by myocardial infarction, and when combined with dobutamine-stress testing, it can identify ischemic myocardium [76,82].

Both imaging modalities require slow resting heart rates at the time of image acquisition, which can be achieved with administration of a beta blocker or calcium channel blocker. Although C-CTA has superior sensitivity to visualize the coronary arteries than MRA, it involves radiation exposure and use of iodine-based contrast agents. Although FDG-PET with myocardial suppression may show inflammatory changes in the proximal coronary arteries, prospective studies to support such a practice are lacking [83,84]. Nevertheless, PET/CT is widely used for assessment of myocardial perfusion in this patient population [85]. Invasive coronary angiography is reserved for patients in whom noninvasive modalities is not feasible, or when coronary revascularization is indicated.

### 2.3. Small-Vessel Vasculitis

#### 2.3.1. Granulomatosis with Polyangiitis

Granulomatosis with polyangiitis (GPA), previously recognized as Wegener’s granulomatosis, is an ANCA-positive multi-system disorder that mainly involves small-sized vessels [86,87]. It is characterized by pauci-immune vasculitis and necrotizing granulomatous inflammation [88]. GPA is a rare disease and mainly affects older adults. GPA predominantly affects the upper and lower respiratory tract as well as the kidneys (pauci-immune glomerulonephritis) [86,89]. Patients typically present with nonspecific constitutional symptoms, and manifestations related to specific organ involvement [89]. Patients with GPA are at increased risk of cardiovascular events including myocarditis, pericarditis, and conduction abnormalities [90].

**Figure 10:**
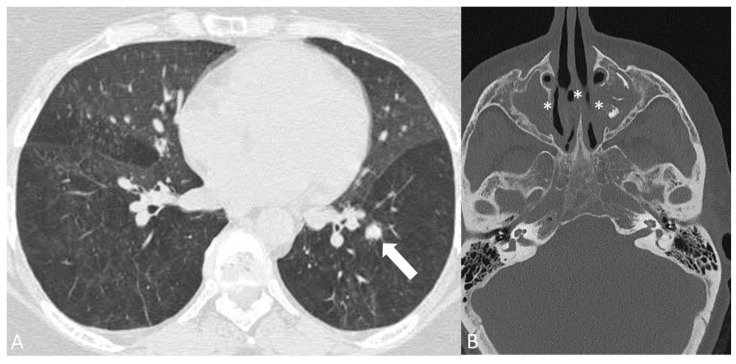
A 48-year-old man with granulomatosis with polyangiitis. CT chest without contrast (**A**) demonstrating a 1.5 cm solid non-calcified pulmonary nodule in the basal left lower lobe (arrow). CT of the facial bones without contrast (**B**) showing diffuse mucosal thickening with near complete opacification of the maxillary sinuses with erosion of the medial wall of the maxillary sinuses and nasal septum (asterisks).

Despite the lack of imaging features specific for GPA, there are known radiological patterns suggestive of the disease [86]. Approximately, 70% of patients with GPA exhibit abnormal findings on chest CT imaging, including pulmonary nodules, cavities, reticulonodular ground-glass infiltrates, and pleural disease [88,91,92]. CT scan also has a superior sensitivity in identifying masses located in the retro-orbital space, paranasal sinuses, and mastoids [93] (Figure 10).

**Figure 11:**
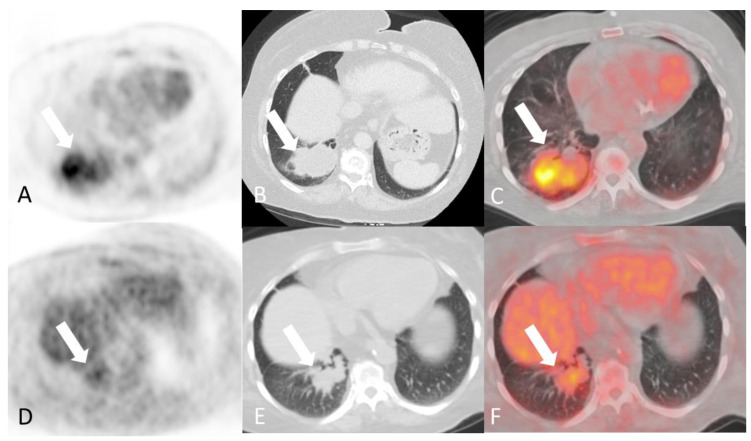
A 50-year-old woman with known granulomatosis with polyangiitis. Axial images of F-18 FDG PET/CT at time of presentation (**A**–**C**) demonstrating a tracer avid mass like consolidative opacity in the basal segments of the lower lobe of the right lung (arrows). Axial images of subsequent F-18 FDG PET/CT approximately 6 months after initiation of immunosuppressive therapy (**D**–**F**) demonstrating known right lung consolidation is smaller and significantly less tracer avid (arrows).

Granulomatous lesions in the lungs, sinuses, or kidneys can be seen on FDG-PET scan and may add additional insights into disease activity [94] (Figure 11). Nonetheless, FDG-PET imaging does not clearly differentiate between vasculitis/granulomatosis, infection, and malignancy [30,95,96].

#### 2.3.2. Eosinophilic Granulomatosis with Polyangiitis

Eosinophilic granulomatosis with polyangiitis (EGPA), previously known as Churg–Strauss syndrome, is a small-vessel necrotizing vasculitis distinguished by peripheral blood eosinophilia and eosinophilic infiltration of affected tissues [97]. EGPA prominently involves the ear, nose, and throat (ENT) region, particularly the nasal passages and paranasal sinuses, leading to chronic rhinosinusitis with and without nasal polyps [97,98]. Cardiac involvement, in the form of endomyocarditis, may lead to severe left ventricular systolic dysfunction, and is associated with high mortality in EGPA [99]

In EGPA, high-resolution chest CT commonly reveals bilateral patchy ground-glass opacities or areas of consolidation, typically distributed in the subpleural regions and lower lobes of the lungs [87]. The role of FDG-PET/CT in EGPA is less well defined, and it is not routinely used to evaluate these patients [100].

### 2.4. Miscellaneous

#### 2.4.1. Behçet’s Disease

Behçet’s disease is a systemic vasculitis of unknown etiology, characterized by recurrent episodes of acute inflammation. It can impact nearly every blood vessel of the body regardless of its size and can potentially affect both arteries and veins [101]. Behçet’s disease is most common in eastern Asia and the Mediterranean area, particularly Turkey. The disease affects men and women equally, predominantly at ages 20–40 years [102]. An association has been found between Behçet’s disease and certain human leukocyte antigens [101]. Clinically, patients present with recurrent episodes of oral aphthous ulcers, urogenital ulcers, and skin lesions [103]. Uveitis is a common ocular manifestation, and neurologic disease may occur in less than 10% of the patients [103].

Vascular involvement is more common in men and considered a major cause of morbidity and mortality [104]. Venous thrombosis commonly manifests as lower extremity deep vein thrombosis, and it can be diagnosed by ultrasound with excellent sensitivity [103]. Other sites include hepatic veins, inferior vena cava, and right atrium. Doppler ultrasound and contrast-enhanced CT or MRI may be used in patients with a suspected Budd-Chiari syndrome related to Behçet’s disease [102].

**Figure 12:**
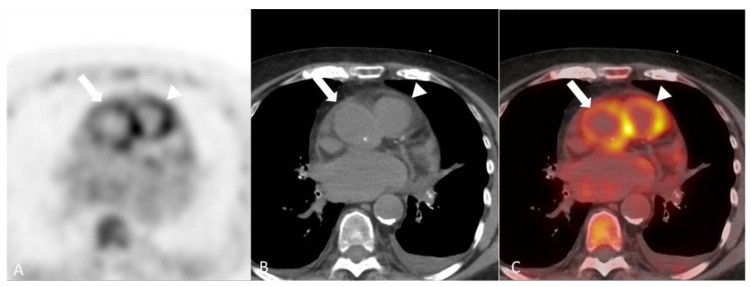
A 75-year-old woman with Behcet’s disease. Axial images of attenuation corrected F-18 FDG PET (**A**), CT without contrast (**B**) and fused PET/CT (**C**) demonstrating concentric tracer avid mural thickening to the ascending aorta involving the root (arrow) as well as the main pulmonary artery (arrowhead), consistent with active vasculitis.

**Figure 13:**
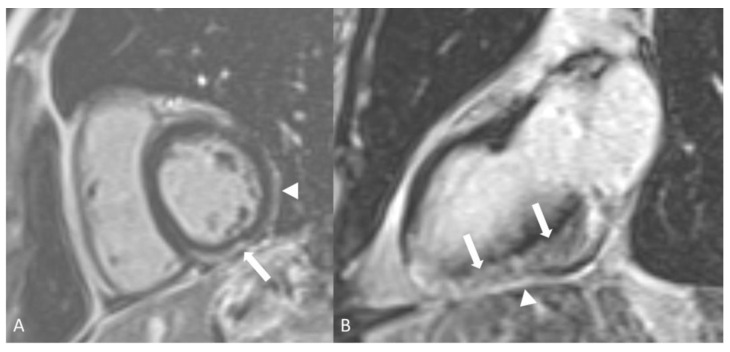
A 53-year-old woman with Behcet’s myopericarditis. Short axis (**A**) and two-chamber (**B**) inversion recovery images of cardiac MR approximately 10 min after intravenous administration of gadolinium demonstrating mid myocardium and epicardium abnormal hyperintense signal consistent with delayed myocardial enhancement in the inferior and inferolateral segments of the left ventricle extending from base to the apex (arrows). In addition, there is delayed enhancement of the pericardium overlying same segments (arrowhead).

On the other hand, arterial disease in the form of arterial wall inflammation is less common and may lead to thromboses, stenoses, and aneurysmal formation (Figure 12). Pulmonary artery aneurysm and/or thrombosis is the most common pulmonary vascular lesion in Behçet’s disease, and pulmonary CT angiography is the preferred method to detect it [102]. Coronary arteritis may occur in Behçet’s disease and be visualized on C-CTA. However, myocardial infarction is uncommon [105]. Myocarditis has been reported in the literature, although very rarely [104]. Cardiac MRI may be used to assess for late gadolinium-enhancement and increased T2 signal when there is a clinical suspicion for myocardial involvement [106] (Figure 13).

#### 2.4.2. Cogan Syndrome

Cogan’s Syndrome (CS) is a rare chronic disorder characterized by ocular and inner ear disease [107]. The pathogenesis of CS is unknown but thought to be related to autoimmunity [108]. Patients mainly present in their twenties with interstitial keratitis and Ménière- like symptoms [109]. Systemic vasculitis can occur in CS including aortitis with aortic root dilatation, aortic insufficiency, or coronary arteritis [109]. Other large vessels may also be involved in a pattern similar to TAK. Although the role of imaging is less clear, CTA and MRA can be used to identify large-vessel involvement in CS [107].

#### 2.4.3. Immunoglobulin G4-Related Disease

Immunoglobulin G4–related disease (IgG4-RD) encompasses multiple different clinical entities that share similar pathological features including an inflammatory phase characterized by IgG4-positive plasma cell-rich infiltrate in the involved organs, and a subsequent fibrotic phase responsible for fibrosis and tissue destruction, if left untreated [110,111]. Diagnosis of IgG4-RD in the early inflammatory phase is crucial as the majority of patients respond to glucocorticoid therapy in this treatment-responsive stage [112].

**Figure 14:**
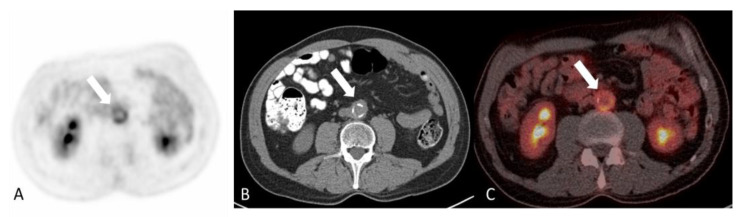
65-year-old woman with IgG4-related aortitis. Axial images of F-18 FDG attenuation corrected PET (**A**), CT without contrast (**B**) and fused PET/CT (**C**) demonstrating tracer avid concentric mural thickening involving the abdominal aorta (arrows) and underlying calcified atherosclerosis. No evidence of retroperitoneal fibrosis.

Vascular involvement occurs in up to 25% of patients with IgG4-RD in the form of aortitis and periaortitis (Figure 14). Abdominal aortitis can be associated with retroperitoneal fibrosis. In fact, impingement of the ureters by fibrosis and subsequent hydronephrosis can be the leading clue to the diagnosis. The radiographic features of IgG4-related aortitis include aortic wall thickening, similar to that described in large vessel vasculitis [113]. Coronary arteritis, leading to large coronary aneurysms or acute coronary syndrome, is increasingly recognized as a manifestation of IgG4-RD [110,111]. Myocarditis is extremely rare [114].

## 3. Conclusions

Diagnosis and management of vascultitic diseases are often challenging and require a multidisciplinary approach due to multi-system involvement. Timely diagnosis is critical as disease progression can be associated with significant cardiovascular morbidity and mortality if treatment is delayed. Advances in multi-modality imaging now enable physicians to visualize areas of inflammation and determine disease activity and extent, allowing both diagnosis and surveillance with treatment. Currently, imaging has become an integral part of the different classification criteria and clinical practice guidelines of these complex diseases, particularly large-vessel vasculitis.

## Figures and Tables

**Table 1:** The advantages and disadvantages of different imaging modality in diagnosis of vasculitis.

Modality	Findings	Advantages	Disadvantages
Ultrasound	Homogenous hypoechoic wall thickening	Reasonable cost Absence of radiation Wide availability	Cannot usually examine deep arteries Operator-dependent
MRI/MRA	Mural thickening Wall gadolinium-enhancement of the affected artery	Can assess cranial arteries and large- vessel involvement. Lack of radiation Ability to depict wall inflammation and complications at a high resolution	Cost Claustrophobia Incompatibility with certain hardware in the body Involves contrast
CTA	Concentric wall thickening Wall enhancement Perivascular fat stranding	High availability, fast Excellent spatial and temporal resolution Coronary CTA can be obtained simultaneously if there is a suspicion for coronary artery disease. May differentiate between vasculitis and atherosclerosis. Can detect stenosis, dissection, or aneurysms	Radiation exposure Contrast complications.
FDG-PET/CT	Increase FDG uptake as a sign of active inflammation	High sensitivity Can assess other pathological conditions (cancer and infection). Assess for active inflammation and monitor response to therapy	Cost Radiation Atherosclerosis may overestimate degree of inflammation.

## Data Availability

Data is contained within the article.
